# Engaging older adults in the process of aging research: a multimethod study evaluating the experience and efficacy of a citizen advisory group for a dementia risk reduction program

**DOI:** 10.1186/s40900-024-00643-6

**Published:** 2024-12-27

**Authors:** Danielle D’Amico, Marie Y. Savundranayagam, Rose Biles, Inbal Itzhak, Nicole D. Anderson, for the Canadian Consortium on Neurodegeneration in Aging (CCNA) CAN-THUMBS UP Study Group, Howard Chertkow, Sylvie Belleville, Howard H. Feldman, Manuel Montero-Odasso, Haakon Nygaard, Nicole Anderson, Sarah Banks, Samir Das, A. Carol Evans, Guylaine Ferland, Joyla A. Furlano, Scott Hofer, Inbal Itzhak, Diane Jacobs, Pamela Jarrett, Andrew Lim, Chris McGibbon, Karen Messer, Carolyn Revta, Julie Robillard, Eric E. Smith, Mark Speechley, Jennifer Walker, Michael Borrie, Kenneth Rockwood, Paul Brewster, Andrew Centen, Marc Cuesta, Senny Chan, January Durant, Tatiana Herold, Nellie Kamkar, Jody-Lynn Lupo, Yanina Sarquis-Adamson, Penelope Slack, Amal Trigui, Shahnaz Winer, Fatim Ajwani, Anas Alrohimi, Nicole Anderson, Geneviève Arsenault-Lapierre, Gabrielle Aubin, Sylvie Belleville, Jennifer Bethell, Louis Bherer, Maude Bouchard, Mark I. Boulos, Jacqueline Burt, Asif Javed Butt, Richard Camicioli, Jennifer Campos, Julie Carrier, Alison ChasteenHoward Chertkow, Rachel A. Crockett, Marc Cuesta, Danielle D’Amico, Véronique Daneault, Thanh Dang-Vu, Alex Desautels, Caroline Desrosiers, Shirley Dumassais, Emily Dwosh, Gillian Einstein, Margaret Fahnestock, Ryan Stanley Falck, Howard H. Feldman, Guylaine Ferland, Alexandra Fiocco, Christine Gagnon, Jean-François Gagnon, Liisa Galea, Aravind Ganesh, Nicole Gervais, Brigitte Gilbert, Nadia Gosselin, Catherine-Alexandra Grégoire, Tatiana Harold, Stephanie Huang, Catherine Hughes, Inbal Itzhak, Raed Joundi, Heather Keller, Elizaveta Klekovkina, Kim Lasnier-Le Quang, Danielle Laurin, Teresa Liu-Ambrose, Marie-Céline Lorenzini, Dominique Lorrain, Kaljani Mahalingam, Sarantia Samantha Maltezos, Brooklyn Mankasingh, Roger Marple, Susan Marzolini, Samira Mehrabi, Paul Mick, Laura Middleton, Tatiyana Mollayeva, Manuel Montero-Odasso, Annabelle Moore, Aline Moussard, Reanne Mundadan, Kelly Murphy, Leigh-Anne M. Noltie, Haakon Nygaard, J. B. Orange, Emilie Parolin, Natalie Phillips, Kathy Pichora-Fuller, Lori Piquette, Jason Plaks, Ron Postuma, Avery Pratt, Maria Natasha Rajah, Helli Raptis, Kayla Regan, Anne Julien-Rocheleau, Marie Y. Savundranayagam, Penelope Slack, Eric E. Smith, Mark Speechley, Kylie Sullivan, Munira Sultana, Fatima Tangkhpanya, Talar Tcherkezian, Anne-Julie Tessier, Lynn Valeyry Verty, Amanda Wagner, Walter Wittich, Stephanie Yamin, Anthonio Zadra, Alan Evans, Samir Das, Rida Abou-Haider, Rolando Acosta, Camille Beaudoin, Charlie Henri-Bellamare, Jessica Callegaro, Milad Heshmati, Zia Mohades, Pierre Pac Soo, Serge Gauthier, Howard H. Feldman, Barry Greenberg, Nathan Herrmann, Krista Lanctot, Joanne McLaurin, Haakon Nygaard, Paul Territo, Donald Weaver, Cheryl Wellington, Danielle Alcock, Quincy Almeida, Sarah Best, Korbin Blue, Isabella Celotto, Lauren Cole, Roger Dixon, Hiroko Dodge, Caroline Duchaine, Nada Elhayek, Thalia Field, Jason Haassenstab, Josée Haché, Andrew Hamilton, Robin Hsiung, Lauren Moniz, Hanin Omar, Chris Pauley, Bryn Robinson, Ève Samson, Jenna Sands, Andrew Sexton, Sheetal Shajan, Sachie Sharma, Mariam Sidrak, Carol Thomas, Julia Truemner, Linda Yetman, Guangyong Zou, Larissa McKetton

**Affiliations:** 1 Rotman Research Institute, Baycrest Academy for Research and Education, 3560 Bathurst Street, Toronto, ON M6A 2E1 Canada; 2 School of Health Studies, Western University, London, ON Canada; 3 Canadian Consortium on Neurodegeneration in Aging (CCNA), Montreal, QC Canada; 4 Departments of Psychology and Psychiatry, University of Toronto, Toronto, ON Canada

**Keywords:** Collaborative research, Co-creation, Citizen advisory group, Evaluation, Program development, Older adults, Multimethod, Brain health

## Abstract

**Background:**

Collaborative research with end-users is an effective way to generate meaningful research applications and support greater impact on practice and knowledge exchange. To address these needs, a Citizen Advisory Group (CAG) of nine older adults (ages 64–80, 67% women) was formed to advise scientists on the development of Brain Health PRO (BHPro), a web-based platform designed to increase dementia prevention literacy and awareness. The current study evaluated if the CAG met its objectives, how inclusion of the CAG aligned with collaborative research approaches, and the CAG’s experience and satisfaction throughout the development process.

**Methods:**

An anonymous online survey was administered to the CAG members and 30 scientist/trainee authors of the BHPro chapters. The CAG also participated in an online focus group.

**Results:**

Most CAG members and chapter authors agreed that the CAG met its primary objectives and added unique value to BHPro. Both groups viewed the CAG’s involvement as well-aligned with engaged scholarship, co-production, integrated knowledge translation, and, to a lesser extent, participatory research practices. CAG members reported high satisfaction with personal goal attainment, which included learning, collaborating with others, and making a meaningful impact. Content analyses of the focus group revealed three categories: 1) personal benefits related to learning, connection, and feeling valued, 2) value of a masked peer-review process, and 3) an accessible final product.

**Conclusions:**

Findings suggest that collaborating with end-users in the process of aging research confers personal and scientific benefits for both older adults and researchers.

**Supplementary Information:**

The online version contains supplementary material available at 10.1186/s40900-024-00643-6.

## Abstract (plain language summary)

When creating a new health program, it is beneficial for researchers to team up with the people who will actually use the program. This collaboration helps make the program better in terms of its quality, how easy it is to use, and how much of an impact it will have. A citizen advisory group of nine older adults helped researchers develop an online program about dementia awareness and risk reduction. This study aimed to understand how these group members felt about their role, whether the group achieved its goals, and how it related to different collaborative research methods. Both the group members and researchers took part in an online survey, and the group members also participated in a focus group interview. The group members enjoyed their involvement and felt that it met their personal goals. They, along with the researchers, believed the group successfully achieved its objectives and added value to the program. They also agreed that the group followed the principles of collaborative research approaches. During the focus group, group members emphasized the personal benefits of participating in the project, their preference for limited direct contact with researchers, and how their involvement made the program more accessible to the public. Overall, when researchers and citizen advisory groups collaborate on health programs, it benefits everyone involved, leading to better program quality and health benefits.

**Supplementary Information:**

The online version contains supplementary material available at 10.1186/s40900-024-00643-6.

## Background

Collaborating with knowledge users in the process of research is an effective means of generating the application of research evidence to real-world problems, promoting a greater change in practice and policy, supporting effective knowledge translation and implementation practices, and bolstering the uptake of evidence-based tools by intended end-users [1]. Collaborative research is especially important in the context of health research, where there is often a disconnect between the creation of scientific resources and use in practice, which ultimately compromises desired health-related outcomes at a population level [2]. Collaborative research aims to narrow the research-practice gap by integrating the needs and preferences of knowledge users throughout the research process to increase knowledge application and use, as well as downstream health outcomes such as subjective cognitive decline [3], suicide prevention [4], physical activity promotion [5], and diabetes management [6].

### Collaborative research approaches

There are various approaches to collaborative research that focus on the co-creation of knowledge and its application to real-world settings [7]. Specifically, *integrated knowledge translation* is a model of collaborative research whereby knowledge users work with researchers to identify a problem and implement research recommendations [8]. Integrated knowledge translation focuses on increasing knowledge use and outcomes and emphasizes an equitable role between researchers and knowledge users throughout the research process. *Engaged scholarship* is a participative research process that focuses on reconnecting academia with societal needs to create knowledge that advances science through leveraging the expertise of both the researchers and stakeholders [9]. *Co-production* refers to the active involvement of consumers in the knowledge production process in order to increase the efficacy of public services, with service users holding the power to improve the planning and delivery of such services [10]. Lastly, *participatory research* involves mobilizing knowledge through collaboration and creates a common understanding of ways to act for the common good, with a specific focus on benefitting underserved/vulnerable communities [11]. Participatory research also focuses on building capacity in communities to foster an equitable role for all groups involved in the research process. Understanding how collaborative research endeavours aligns with the guiding principles of these collaborative research frameworks is important in order to facilitate clarity in communication amongst researchers and knowledge users regarding expectations and the structure of the working relationships [7].

One approach to collaboration with end-users in the research process is through the use of citizen advisory groups. Citizen advisory groups consist of a group of stakeholders from a particular community intended to provide feedback and advice on a research project. Previous research has shown that citizen engagement improves knowledge user experience and health outcomes [12, 13]; is associated with an increase in self-confidence, skill building, and satisfaction among advisory group members [14]; and broadens the perspectives of researchers working with citizen advisory groups [15]. To address the need for collaborative research, health funding agencies in Canada have promoted funding and capacity-building through research partnerships to improve knowledge use and cultivate optimal health outcomes [16]. In response to these needs, a Citizen Advisory Group (CAG) was formed to advise researchers on the development of a national dementia risk reduction program, Brain Health PRO (BHPro).

### The Canadian consortium on neurodegeneration in aging brain health PRO program

BHPro is a 12-month, web-based educational program designed to improve dementia literacy, promote brain-healthy lifestyle changes, and reduce the risk of dementia amongst cognitively intact older adults and individuals with mild cognitive impairment [17, 18]. The program contains eight modules, with the first module providing an introduction about healthy aging and various forms of dementia, and the other seven modules discussing a separate modifiable dementia risk factor domain (diet and nutrition, physical activity, cognitive engagement, sleep, vascular health, psychological and social health, and sensory health). Participants are provided with a personalized dementia risk profile in order to set personal goals related to dementia risk reduction and healthy lifestyle changes. Each module is composed of ~10-min interactive chapters, professionally narrated in English and in French, which provide detailed information on sub-topics related to the module theme (e.g., the importance of managing stress for psychological and social health). BHPro will be made available for free to the general public in Canada and will be used as part of randomized clinical trials in Canada to reduce dementia risk and prevalence. BHPro was developed and authored by 181 dementia researchers across Canada who are members of the Canadian Consortium on Neurodegeneration in Aging (CCNA), a network of researchers, clinicians, and trainees dedicated to accelerating progress in research related to the prevention, treatment, and management of age-related neurodegenerative disease. The BHPro CAG was formed to provide the authors with feedback on the content clarity and level of engagement of the chapters in order to promote acceptability, efficacy, accessibility, and uptake of the program amongst the intended end-users.

## Study Objectives

In addition to the formation and inclusion of a citizen advisory group, current guidelines to establishing effective citizen engagement also recommend *reflection* as integral part of the collaborative research process through feedback from both the researchers and knowledge users [19]. In line with these recommendations, the objectives of the current multimethod study were to evaluate whether the BHPro CAG met its intended objectives and to determine how inclusion of the CAG aligned with collaborative research approaches (i.e., integrated knowledge translation, co-production, engaged scholarship, and participatory research) using feedback from the CAG members and BHPro chapter authors. This study also sought to understand the experience and satisfaction of the CAG members through their involvement in the research process. The GRIPP2 (Guidance for Reporting Involvement of Patients and the Public) checklist [20] was followed and completed (see Supplementary Table 1). Given the exploratory nature of the study, no a priori hypotheses were generated.

## Methods

### Participants

Participants in the current study included the nine BHPro CAG members, as well as 30 scientists and trainees (i.e., Master’s, PhD, PhD/MD, postdoctoral researchers) who authored the BHPro chapters. The CAG was formed in October 2019, and members were recruited by CCNA scientists through their networks. The membership of the CAG was constructed to reflect the intended end-users of BHPro. The CAG members resided in Ontario, Quebec, and New Brunswick, and had English or French as a first language. Table 1 shows the demographic characteristics and volunteer history of seven of the nine CAG members who completed the demographic questionnaire (two CAG members declined to complete the demographic questionnaire). As group meetings were held virtually using Zoom, members were required to have access to a computer and have sufficient computer literacy in order to attend meetings and review digital files. A chair and co-chair of the CAG were also appointed by author NDA to lead group meetings and act as the primary channel of communication between the CAG and the BHPro staff and scientists.

**Table 1:** Demographic characteristics and volunteer history of the CAG members (*n* = 7)

Characteristic	% (*n*)
Age in years (range)	64–80
Gender (%)	
Women	57 (*n* = 4)
Men	43 (*n* = 3)
Ethnicity (% Caucasian)	100 (*n* = 7)
Retired (% yes)	100 (*n* = 7)
Educational attainment (%)	
Some post-secondary education	28 (*n* = 2)
Master’s degree	57 (*n* = 4)
Professional degree	15 (*n* = 1)
Primary language	
English	50 (*n* = 4)
French	50 (*n* = 4)
Hours committed to the CAG (range)	162–1000
Volunteered outside of the CAG during last 5 years (%)	85 (*n* = 6)
Hours/month volunteered before COVID-19 (range)	3–30
Hours/month volunteered after COVID-19 (range)	0–8

The primary responsibility of the CAG was to advise authors on the BHPro chapters using a set of predetermined, approved guidelines (see Table 2) assessing the clarity of the chapters, whether the language was appropriate for the intended audience, if specific actions were encouraged, and if the messaging was encouraging and realistic. These guidelines were developed and agreed upon by the BHPro study team and the educational technology company that was hired to design the program. The CAG members were instructed not to comment on grammar, spelling, length of the chapters, or any graphics that the authors included as these would be corrected by BHPro staff.

**Table 2:** BHPro review guide that the CAG used to assess individual chapters of BHPro

Review guideline items
1. What are the key takeaways of the chapter?
2. What are the points to support the key takeaway?
3. What are the action(s) encouraged?
4. Is the takeaway clear at the outset?
5. Are points given to support key takeaways?
6. Was the chapter understandable?
7. Were there any unfamiliar concepts which were not adequately explained? Give details
8. Did the chapter achieve its goals as set out in the key points outline?
9. Other comments
10. Optional comments on individual slides

Responses to each item were open-ended

The review process was facilitated by the CAG chairs and the BHPro project coordinator. The scientists and trainees who authored the chapters sent their chapter drafts to the BHPro project coordinator, who first conducted a preliminary review and then forwarded the files to the CAG chairs, who then sent it to the three CAG members assigned to review the given chapter. One of the three reviewers was assigned as the note taker to record comments on the chapter review document. While chapter authors were not required to incorporate all of the CAG feedback, a knowledge translation practitioner was available to support the CAG and chapter authors as needed and helped to facilitate implementation of the feedback to authors. Upon completion of the review and once all reviewers agreed on the written comments, the chapter reviews were sent back to the project coordinator who forwarded them to the chapter authors. The CAG members who reviewed the chapters provided their names on each review document that was sent to the authors. The chapter authors were listed in the chapters that the CAG members reviewed, although in many cases the CAG members did not know the authors. The CAG also held biweekly team meetings to discuss progress, points of interest, and address any questions or concerns. The CAG members decided on the frequency of team meetings amongst themselves at the outset of their involvement. All CAG members were invited to attend as many BHPro team meetings as possible with the BHPro experts, which included scientists and staff. Each member of the CAG received $160 CAD quarterly to cover costs (e.g., paper, toner, Professional Zoom licenses) and in appreciation for their time and effort. This study was approved by the Baycrest Research Ethics Board (REB 22-02).

### Online survey

The online survey was created by NDA, DD, II, and one CAG member. Both the CAG and the BHPro chapter authors completed the survey through REDCap electronic data capture tools [21, 22] hosted at the Rotman Research Institute. The survey consisted of 28 Likert-type questions assessing the degree to which the CAG met its intended objectives (see Supplementary Table 2) and how inclusion of the CAG aligned with collaborative research approaches (see Supplementary Table 3) from 1 (*strongly disagree*) to 5 (*strongly agree*). Nine items asked about whether the intended objectives of the CAG were met. These items included: advising on the overall plan of BHPro, reviewing material and providing feedback on the program chapters, and assessing whether the chapters accomplished their stated goals. The items also included: assessing the clarity and language suitability of the chapter information, evaluating whether the chapters encouraged specific actions and if they were realistic for the intended audience, and advising on the implementation and methods of recruitment for the BHPro. The additional 19 items evaluated the alignment between the inclusion of the CAG and the guiding principles of integrated knowledge translation (5 items), participatory research (8 items), engaged scholarship (4 items), and co-production (3 items). One item corresponded to both integrated knowledge translation and participatory research. All items were derived from Nguyen et al. [7]. Two open-ended questions were also included, which asked participants about the best part of the CAG’s role and what could be improved. These items were added to the survey to aid the development of the focus group interview questions.

An additional set of 37 questions were provided to the CAG to better understand their satisfaction with other aspects of their involvement (see Supplementary Table 4). These items inquired about personal experience, their satisfaction with the organization and structure of the CAG, and their experience attending the CAG team meetings and the expert team meetings with the scientists. Participants rated each item from 1 (*strongly disagree*) to 5 (*strongly agree*) or 1 (*very dissatisfied*) to 5 (*very satisfied*), depending on the question. The CAG members were also asked to provide up to three of their top goals for participating in the CAG (one goal was required), as well as the degree to which they felt that each goal was met on a scale from 0 (*not met at all*) to 100 (*exceeded my expectations*).

One of the CAG chairs collaborated with DD and NDA to develop and refine the survey items, and subsequently did not complete the online survey.

### Focus group interview

An online focus group using a semi-structured interview guide was conducted via Zoom after completion of the online survey with eight of the CAG members and facilitated by DD to gain a more in-depth understanding of their experience as CAG members. The CAG member who declined to participate in the focus group was a different CAG member than who collaborated on the survey development. The interview questions were derived based on the objectives of the current study pertaining to CAG members’ experience working with scientists and the degree to which their objectives were met. We also focused on topics which appeared to have less consensus amongst CAG members and between the CAG and the chapter authors related to generalizability of Brain Health PRO to underserved communities. Questions inquired about CAG members’ experiences working with the chapter authors, ways their engagement could have been improved, potential modifications to the program to better serve diverse communities, any overlooked lived experiences within the CAG membership, and any other aspects of the process where they desired increased involvement. See the Supplementary Material for the focus group interview questions. The focus group was approximately 90 min in length and was recorded and transcribed using Zoom’s recording feature, with the transcription checked and corrected by a staff member.

### Data analyses

#### Quantitative

Responses on the items corresponding to each of the four collaborative research approaches (i.e., integrated knowledge translation, participatory research, engaged scholarship, and co-production) were averaged to create an overall score for each collaborative research approach. The item that was aligned with both integrated knowledge translation and participatory research was included in the overall score for both approaches. To determine which collaborative research approach best aligned with the inclusion of the CAG, Friedman’s tests were conducted on the overall scores separately for CAG members and chapter authors with a statistical significance threshold of *p* < 0.05. Wilcoxon signed-rank tests with a Holm-Sidak adjustment for multiple comparisons were conducted to evaluate pairwise comparisons among the four collaborative research approaches separate for CAG members and chapter authors. All other quantitative results are reported using descriptive statistics.

#### Qualitative

Conventional content analysis [23] was used to analyze the focus group data. The goal was to explore the experiences of the CAG members and their perspective on the collaborative research approach. Two researchers (MIS and RB) used an inductive method approach by identifying recurrent words and codes from the transcript, and grouping them into similar themes [23, 24]. Discrepancies between codes and themes generated were discussed until a consensus was reached. Member checking was completed by sharing the categories and getting feedback from participants in a follow-up meeting.

## Results

### Survey findings

#### CAG members

**Fig. 1:**
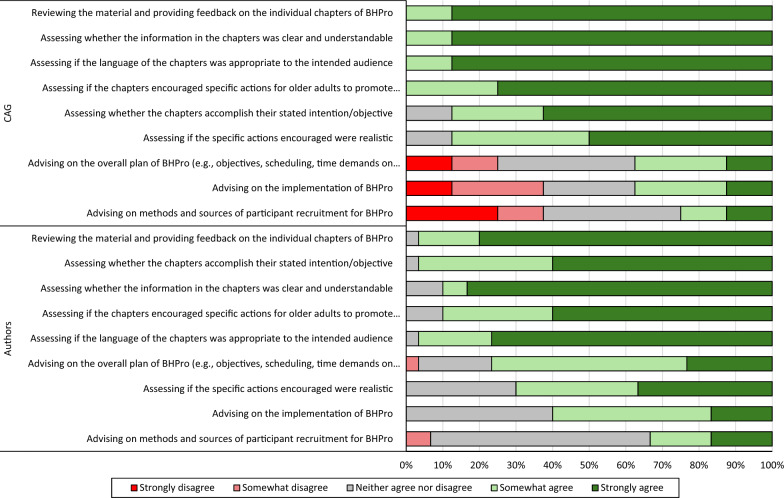
The proportion of CAG members (top) and chapters authors (bottom) who agreed that the CAG met their intended objectives. (BHPro: Brain Health PRO; CAG: Citizen Advisory Group)

Eight of the nine CAG members completed the survey. As depicted in Fig. 1, when asked about the degree to which the intended objectives of the CAG were met, all eight members agreed that the CAG reviewed and provided feedback on the BHPro chapters, assessed the clarity of the chapters, evaluated the suitability of the chapter language, and assessed whether the chapters encouraged specific actions. Seven out of the eight CAG members agreed that the CAG assessed whether the chapters accomplished their objectives and if the specific actions encouraged by the chapters were realistic. Three CAG members agreed that the CAG met the objective of advising on the overall plan of BHPro (*n* = 2 disagreed) and advising on the implementation of the program (*n* = 3 disagreed). Two individuals agreed that the CAG advised on participant recruitment for the program, while three disagreed with this intended objective.

**Fig. 2:**
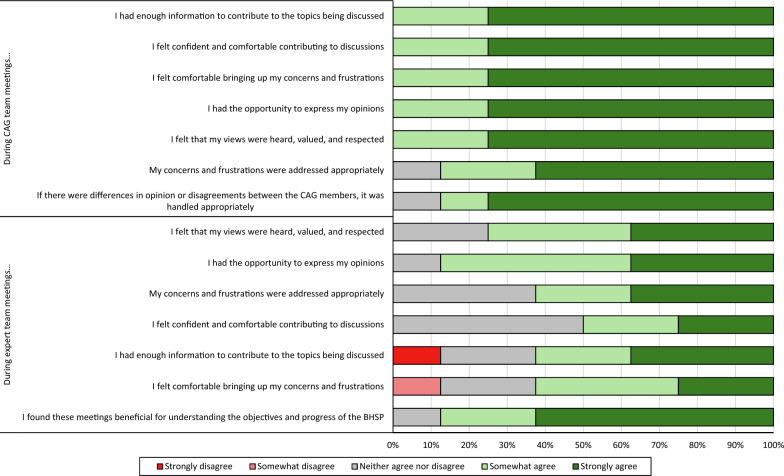
The percentage of CAG members who were satisfied with the CAG team meetings (top) and expert team meetings (bottom). (BHPro: Brain Health PRO; CAG: Citizen Advisory Group)

As shown in Fig. 2, when asked about the biweekly CAG team meetings, seven to eight members of the CAG agreed that they felt comfortable and had enough information to contribute to discussions (eight members), felt comfortable raising concerns (eight members), they were provided the opportunity to express their opinions (eight members), their views were valued and respected (eight members), that their concerns were addressed appropriately (seven members), and disagreements were handled appropriately (seven members). When asked about the biweekly expert meetings, four to seven individuals agreed with the aforementioned sentiments, depending on the statement (see Fig. 2). One individual disagreed that they had enough information to contribute to discussions during the expert meetings, and one individual disagreed that they felt comfortable raising concerns. Seven individuals found the expert meetings beneficial for understanding the objectives and progress of BHPro.

**Fig. 3:**
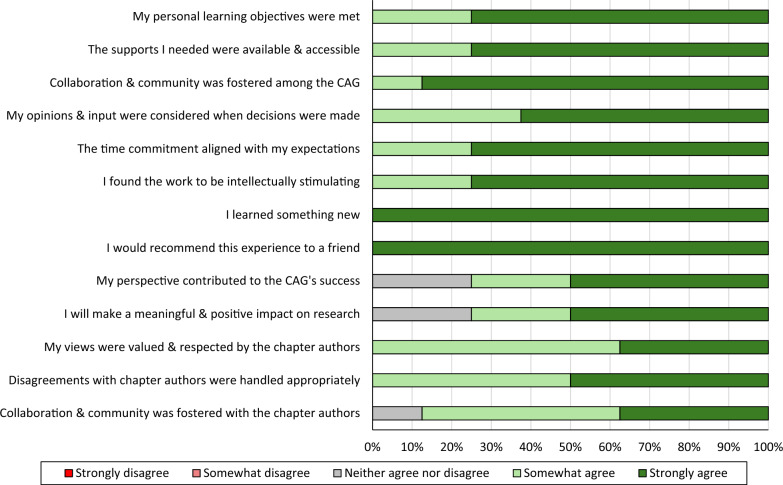
Proportion of CAG members who agreed with different aspects of their involvement in the development of Brain Health PRO (CAG: Citizen Advisory Group)

All eight CAG members felt that their personal learning objectives were met, supports were made available in order to succeed, a sense of community was cultivated amongst the CAG, their opinions were considered in decision making processes, and the time commitment was within their expectations. All eight CAG members also agreed that the work was intellectually stimulating, they learned something new during the experience, their views were heard and valued by the chapter authors, that disagreements with the chapter authors were handled appropriately, and that they would recommend their involvement with the CAG to a friend. Seven CAG members felt that a sense of community was fostered with the chapter authors, and six members agreed that their unique perspectives contributed to the success of the CAG and would make a positive effect on research in brain health and dementia risk reduction. See Fig. 3.

**Fig. 4:**
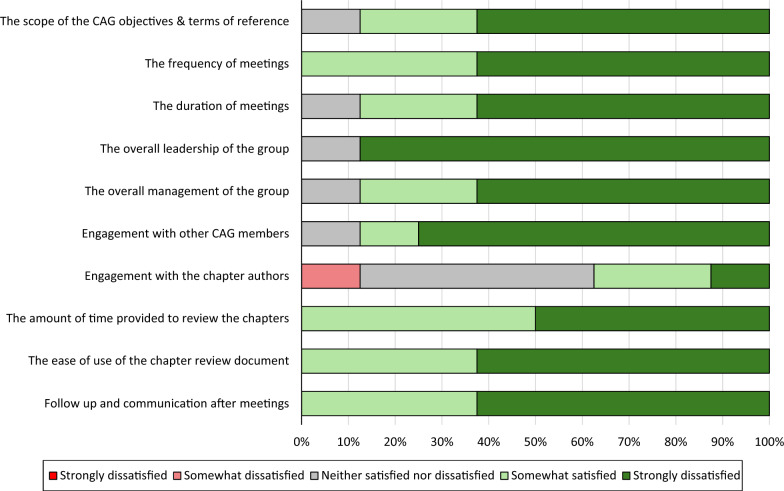
The proportion of CAG members who were satisfied with different aspects of the organization and structure of the CAG. (CAG: Citizen Advisory Group)

When asked about the organization and structure of the CAG, all eight participants were satisfied with the frequency of meetings, the amount of time allotted to review chapters, the use of the review guideline document, and follow-up communication outside of meetings. Seven out of eight CAG members were satisfied with the overall scope of the CAG, the duration of meetings, group leadership and management, and engagement with other CAG members. Three individuals were satisfied with the engagement with the chapter authors, while one individual was dissatisfied with chapter author engagement and the four remaining participants indicated that they were neither satisfied nor dissatisfied. See Fig. 4.

**Fig. 5:**
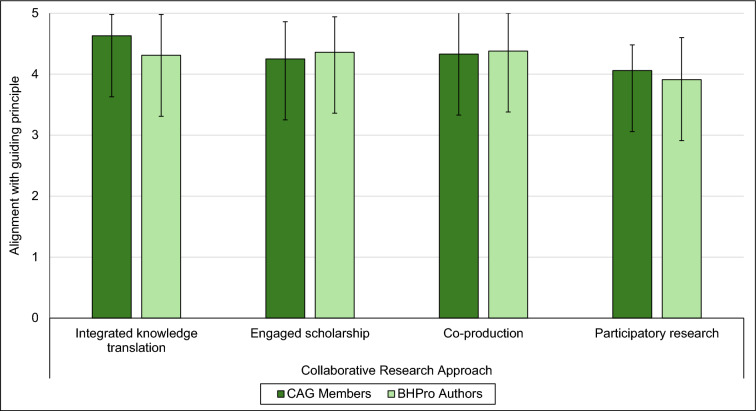
Average ratings for each collaborative research approach among CAG members and BHPro chapter authors. (BHPro: Brain Health PRO; CAG: Citizen Advisory Group

Among the CAG members, there was a statistically significant main effect of collaborative research approach alignment with the inclusion of the CAG in the development of BHPro (*χ*^*2*^(3) = 8.51, *p* = 0.037). However, post hoc analyses did not reveal statistically significant differences among the collaborative research approaches. See Fig. 5 for the average ratings on each collaborative research approach among CAG members and chapter authors.

The CAG members endorsed personal goals for their involvement in the CAG pertaining to contribution to the advancement of knowledge, acquiring their own knowledge about a new subject area, collaborating with others, and optimizing the user experience of BHPro. Seven individuals endorsed three goals, and one individual endorsed two goals. Overall, the CAG members were satisfied with their personal goal attainment, with an average score across all goals of 85.18 out of 100 (SD = 10.44, range = 66–100).

#### Chapter authors

A total of 30 out of 181 chapter authors (22 scientists, 8 trainees) completed the online survey, of which one scientist and one trainee did not provide responses for the questions pertaining to alignment with collaborative research approaches.

Ninety percent to 96.7% of respondents agreed that the CAG met their intended objectives of reviewing and providing feedback on the BHPro chapters, assessing whether the chapters accomplished their objectives, assessing the clarity of the chapters, determining whether the chapters encouraged specific actions, and evaluating the suitability of the chapter language. A smaller proportion of the chapter authors agreed that the CAG met their objectives of advising on the overall plan of the project (76.7%), assessing if the specific actions encouraged by the chapters were realistic (70%), and advising on the implementation of the program (56.7%). A total of 33.3% of chapter authors agreed that the CAG advised on program recruitment. See Fig. 1.

Among the chapter authors, there was a statistically significant main effect of collaborative research approach alignment with the inclusion of the CAG in the development of BHPro (*χ*^*2*^(3) = 22.95, *p* = < 0.001). Post hoc analyses revealed that participatory research was rated as the least-aligned compared to co-production (*Z* = − 4.13, *p* < 0.001), engaged scholarship (*Z* = − 3.78, *p* < 0.001), and integrated knowledge translation (*Z* = − 3.85, *p* < 0.001). There were no statistically significant differences among co-production, engaged scholarship, and integrated knowledge translation. See Fig. 5.

### Focus group findings

The following three categories emerged from the qualitative analysis: (1) personal benefits, (2) value of a masked peer-review process, and (3) accessible final product.

#### Personal benefits

Being a part of the CAG offered members enjoyable opportunities for learning, teamwork, social connections, and meaningful experiences. Several participants shared that the CAG activities allowed them to learn and grow, which was important to them. Participants had varying degrees of experience in healthcare and/or knowledge about health. The new learning included content about brain health but it also included learning to work as a team to offer constructive feedback to scientists. This was exemplified by the following quotation:

I think both the challenge of the new learning, but also the challenge of containing ourselves was a great learning experience for all of us as we go forward into hopefully other exciting projects, but, what a team. (CAG Member #7).

It is important to note that the CAG began online via Zoom before the COVID-19 pandemic. This was fortuitous as the group continued its operations when physical distancing was required and/or encouraged during the pandemic. Participants reported that being connected socially through this online network helped them throughout the pandemic. One participant noted:

I was finding myself quite isolated and quite anxious through the pandemic. And this gave me a focus, which was sorely needed at that point. (CAG Member #8).

Another participant reported enjoyment in the combination of the topic itself and the contact with *members from across the country: *

I really enjoy that experience, the project itself, but we have to realize we were most of it in the middle of a pandemic as well and in confinement for parts so that was a contract with many different people across different places in the country and different expertise in backgrounds. So, not only was a topic in itself interesting and engaging in what we were receiving, but the people were quite something else. And so, I truly enjoyed it. (CAG Member #3).

Participating in the CAG offered meaningful engagement for its members. Participants felt heard, respected, and appreciated by project leaders and scientists, as reflected here:

when I was first approached to do this, I was sceptical because I thought, why are 60 high powered scientists gonna listen to a bunch of older folk with no kind of background? And, and so I was always sort of the voice of scepticism in this, but I was amazed, constantly amazed, first of all, by the support that we got from [lead scientists] and that they actually listened to us. And, and this to me was amazing. Like, it really exceeded my expectations in that way. Because of that, I feel that, instead of being, um, a frill…it was very meaningful. I think that’s really, I think that we really played a key role. I think that everybody did an absolute bang-up job. It was great. (CAG Member #6).

This quotation highlights that CAG members’ contributions were taken seriously and that they felt a sense of meaningful engagement and impact through their involvement.

#### Value of a masked peer-review process

Members of the CAG appreciated that the peer-review process was independent of direct collaboration with chapter authors, which allowed for open feedback. This was reflected in the following statement:

There were no personalities involved. When we looked at a chapter, it was the chapter. It wasn’t somebody that you had had to speak to directly about the content. So, I think in some ways, the non-relationship worked very well in our favour. (CAG Member #7).

Participants reported that it was challenging to respond to the work of chapter authors with different writing styles and perspectives. However, the process was eased as the feedback process was devoid of egos or competing personalities, as indicated by the following quotation:

We were commenting on a text, so we had complete freedom. And I think that is important. It allowed us to do our job, the best we could because there were no egos. There were no personalities, there was nothing at all. And I think that should be preserved in, in the future for any of these things. (CAG Member #3).

Participants commented that the review process itself became more streamlined by including a project coordinator and creating clear onboarding instructions, which helped with the process, especially for chapters that included too much technical jargon. Participants commented that the chapter authors have a lot of knowledge but were not always able to distil the key points in an accessible way. Having the project coordinator give initial feedback to authors supported the efficiency of the review process. Second, the CAG created an onboarding template for chapter authors to ensure that key points were included in their chapters. Specifically, they created

boundaries…so that they [scientists] understood that the takeaways for the end-user were critical when they were designing their chapters. And I think that made a huge difference again, in the evolution of what we received. (CAG Member #7).

#### Accessible final product

Finally, the CAG members recognized that a group reflective of the end-user ensured its accessibility to the public. As mentioned in the previous section, CAG members were focused on the quality of writing as it pertained to the target audience, as shown in this quote:

You’re writing to the general for the general public, and there are ways, and there are words that you don’t use, you know, the long technical words. (CAG Member #3).

In addition to the quality of the writing, they also helped the authors distil their messages into actionable items, as evidenced by the following quote:

Originally, they were just submitting chapters and they were really all over the map. So when we narrowed them down a little bit where they had to talk about key takeaways and whether their goal in the chapter was met by the answers to those key takeaways, that that helped a lot… It was probably a learning experience on the expert side also. (CAG Member #7).

The CAG members noted that their perspective and birds-eye view of the entire program allowed them to provide important feedback to the authors. Indeed, one CAG member mentioned that

because we did many, many, many chapters…on different topics, we had a better overview than individual scientists who were doing their own chapter. And like, we picked up on a number of things…I remember…two different chapters…and we said…this place here, and this place here, you’re contradicting each other. So you need to figure this out. And they did.” (CAG Member #3). Participants were mindful about their important role in having an end-product that would be used by the public. This was evidenced by the following quote: “Right from the outset was to ensure that what we received was the best that it could be for the end-user. And we were representing the end-user. (CAG Member #7).

Another participant commented on the crucial role of the group in ensuring the final outcomes would benefit older adults:

I think it shows the value of bringing a group like us on board to really make sure that the end result is for the seniors…we played a major role in that [laughs]. I laughed because left to their [authors] own devices, I could not event imagine what these chapters would have looked like. I think without CAG, this could never succeed. (CAG Member #5).

## Discussion

Engaging with end-users in the research process is important for generating relevant application of research evidence, supporting effective knowledge exchange, and narrowing the health research-practice gap. To address these needs, a CAG of older adult members was involved in the development of BHPro, a web-based dementia literacy and risk reduction program, to advise researchers on the relevance and applicability of the program. In line with the guidelines on effective citizen engagement [16], the purpose of the current study was to gather feedback from the CAG members and BHPro chapter authors on whether the CAG met its intended objectives, and the CAG members’ satisfaction with their experience. This study was also the first, to our knowledge, to understand how a citizen advisory group aligns with collaborative research approaches (integrated knowledge translation, co-production, engaged scholarship, and participatory research). Survey findings showed that, overall, the CAG and chapter authors agreed that the CAG’s objectives were met, the inclusion of the CAG was well-aligned with all four collaborative research approaches, and the CAG was satisfied with their experience and personal goal attainment related to learning, collaboration, and contribution to a worthy cause. Focus group findings with the CAG members revealed three key categories: personal benefits related to learning, connection, and feeling valued; the value of a mask peer-review process with the BHPro chapter authors; and the importance of the CAG in generating an accessible final product that is relevant for the public.

The CAG members generally agreed that the CAG met its intended objectives. This is not surprising as the objectives of the CAG directly mapped onto a preestablished set of guidelines and the BHPro chapter review document that was created at the inception of the CAG by the members with input from a scientist that liaised between the CAG and the chapter authors. The CAG objectives also echo the chapter review guide that the CAG used to assess the individual chapters of BHPro, which was also developed before the CAG began reviewing chapters and sending feedback to the authors via the project coordinator. This is also true of the overall positive ratings from the CAG members pertaining to their satisfaction with the CAG protocol and parameters, which were largely drawn from the CAG terms of reference document that was developed and agreed upon by all CAG members before their work began. Together, these findings emphasize the importance of creating CAG guidelines and parameters before the project begins so that objectives are clear, leading to greater satisfaction and a better experience.

The findings on the attainment of the CAG objectives were also positive among the chapter authors, providing additional evidence that the CAG succeeded in meeting its intended objectives. It should be noted that the objectives that were rated the lowest among both groups pertained to other aspects of the BHPro development that extended beyond reviewing the chapters, specifically advising on program implementation, participant recruitment for piloting and participating in the program, and the overall study plan. This is likely due to the fact that program implementation and participant recruitment was intended to happen while the CAG was involved in the project, but the timeline was delayed, which prevented the CAG from advising on these aspects of the program.

Another key aspect of the current study objectives was to evaluate how the inclusion of the CAG aligned with four key collaborative research approaches: integrated knowledge translation, co-production, engaged scholarship, and participatory research. While these approaches are similar in their overarching goal of co-creating knowledge that is applicable to end-users in real-world settings, they vary with respect to their specific purpose, primary motivation, and perspectives on the power sharing dynamic between researchers and knowledge users involved the co-creation process [7]. It is important to understand how collaborative research efforts, such as inclusion of citizen advisory groups, are aligned with collaborative research approaches in order to establish expectations of the structure of the working relationships during the research process. While the parameters of the CAG and the structure of the working relationship between the CAG and the chapter authors were not built upon a specific collaborative research approach at the outset of the project, it is still worthwhile to understand how the BHPro CAG aligned with the approaches in order to identify successes to build upon and potential pain points to work through in future research. Overall, ratings from the CAG members and chapter authors were relatively high across all four collaborative research approaches, with mean ratings ranging from 4.06 to 4.63 among CAG members and 3.91 to 4.38 among chapter authors out of a total possible score of 5. There were no significant differences, however, across the four approaches among CAG members. This could be due to the small sample size limiting the statistical power to detect true differences, or that the CAG believed that their involvement aligns equally among and taps into the guiding principles of each of the four approaches.

The chapter authors rated participatory research as the least aligned with the inclusion of the CAG compared to integrated knowledge translation, co-production, and engaged scholarship. In inspecting the ratings for the individual items comprising the participatory research score, the items related to BHPro benefitting and meeting the needs of underserved/vulnerable populations were rated the lowest both within the participatory research approach and across the items in the other approach scores. These views are supported by responses from two authors to the open-ended questions on the survey stating,

Although easier said than done, aim to include people of lower socioeconomic status as they are those most likely to benefit the most from such a program., and I said that I disagreed to the statement that the program will be helpful to those in vulnerable communities. I said this because I’m not sure how accessible this information is - whether older adults in these communities have access to computers and the internet, whether they even know about the program, whether the program can be offered in languages other than English or French.

In the focus group, a CAG member also mentioned:

I think, people understand the value of the program, but if you don’t have a computer or a phone or an iPad, you’re not gonna be able to access it. (CAG Member #5).

Although the CAG members were diverse in terms of age range, gender (i.e., approximately equal number of men and women), and occupational background, they were all Caucasian, well-educated, spoke English and/or French only, and mostly resided in Ontario or Quebec with two members residing in an area of New Brunswick with Internet access. This does not reflect the sociodemographic landscape of Canada’s aging population. Diversity of the CAG could be improved by including individuals with lower educational attainment, different racial and ethnic backgrounds, and from more rural areas where access to Internet and technology can differ from urban settings. However, bringing together citizen advisory groups from diverse geographic locations requires a virtual component (e.g., group meetings via Zoom). It is therefore important for a study team to provide Internet and computer/tablet access to those without to promote inclusion among technologically-isolated individuals. It is also important to incorporate a sex and gender lens to understanding the experience and efficacy of future citizen advisory groups with a larger group size. Of note, information about chapter authors’ sociodemographic backgrounds, field of study, and prior experience working with end-users in research were not gathered, preventing any conclusions from being drawn about how their backgrounds influence their perceptions of the CAG’s involvement in the project. Together, these findings highlight the importance of equity, diversity, and inclusion considerations when co-creating health and educational research tools by ensuring that the CAG best represents the end-user.

The differences in collaborative research approach ratings among the CAG may be more apparent in the focus group findings. Although not statistically significant, integrated knowledge translation had the highest average rating among each collaborative research approach. The purpose of integrated knowledge translation is to increase the chances that research findings will be applicable to the end-user (i.e., community-based older adults without dementia in the context of BHPro), with the primary motivation of increasing knowledge (i.e., about dementia prevention and brain health) and driving behaviour change (i.e., the uptake of healthy lifestyle behaviours). This aligns with the focus group category of creating an accessible end project. Indeed, the CAG members highlighted that their role was important to ensure that the chapter authors’ content was accessible, relevant, and ultimately the most beneficial for the end-user. Preliminary data from BHPro pilot studies have, indeed, shown high rates of participant satisfaction and program completion [28]. Conversely, the CAG members rated participatory research as the least aligned with the inclusion of the CAG, with the item stating the CAG had equal or equitable power throughout the development process rated the lowest within the participatory research score, likely driving these findings. The focus group category of the importance of a masked review process may provide additional context. Indeed, the CAG members noted that they valued the lack of direct collaboration and communication with the chapter authors, and instead appreciated that feedback was provided to the authors via a third party. They further remarked that they appreciated this working relationship as it provided them an open opportunity to give honest, but anonymous, feedback to individuals with different skill levels in writing about scientific research for a lay audience. These findings are striking, as the survey results from the CAG members suggest that they were, in fact, dissatisfied with engagement with the chapter authors and their experience attending the expert team meetings. These contrasting findings may be explained by misinterpretation of the survey items, whereby the focus group provided additional context to their answers. It should be noted that attendance at the expert team meetings, the only opportunity to engage directly with scientists, was optional for CAG members, and so the findings may differ depending on meeting attendance, which was not asked about.

In examining the chapter author survey feedback, it appears as though the chapter authors did not share the same sentiment as the CAG about a masked peer-review process. In the open-ended survey questions, eight of the 17 responses to suggestions for improvement mentioned that they would have liked more direct communication with the CAG throughout the chapter development process.

It would help to have more direct communication with a member of CAG for some of the chapters to ask for clarification on some of the initial feedback., I wish there had been an easier way to "rebut" or discuss some of the CAG requested changes. Such as a Zoom call.

This is in direct contrast with the CAG, who believed that the purpose of their role in the BHPro development and the benefit that they provided (i.e., ensuring accessibility of BHPro for the end-user) does not require direct communication with the authors. This is in line with a previous study that sought to understand the impact of collaborative research practices among researchers, who reported that they appreciated having face-to-face contact with advisors, as opposed to exchange of documents only [15]. Future work in this area must be grounded in an approach that can balance the power differentials of CAG members and researchers (e.g., through masking, greater use of a knowledge translation practitioner’s expertise), while also giving the option for chapter authors to glean greater insights from the CAG should they require additional information or have clarification questions that can enhance the quality and accessibility of their work. The CAG’s volunteered time, talent, and resources must also be considered through this process.

The findings from this study also show that the inclusion of the CAG conferred personal benefits for the CAG members. Indeed, the CAG endorsed personal goals of contributing to the advancement of scientific knowledge, acquiring their own knowledge in a new subject area, collaborating with others, and ultimately optimizing the end-user experience of BHPro. Importantly, their satisfaction with goal attainment was very high, indicating that their personal goals for participating in the CAG were met or exceeded. Building on this, overwhelmingly positive findings were also found for their personal satisfaction as a CAG member related to these goals. The focus group findings showed that the group especially appreciated the social connection they built and teamwork they fostered while working together, which was especially important during the COVID-19 lockdowns when they were feeling socially isolated. The CAG members also emphasized that they appreciated feeling valued through their contribution, which enhanced their sense of meaningful engagement. These findings are aligned with previous studies of citizen advisory group members showing that participation in a citizen advisory group is a meaningful experience, socially and intellectually [14, 29]. Together, these findings suggest that the benefits of the CAG extend beyond BHPro itself, but also confer benefits for the older adult CAG members by providing them a unique and meaningful opportunity to contribute to the advance of scientific knowledge on dementia prevention and brain health, learn new skills, and socially engage with others. Cognitive and social engagement that can be achieved through volunteering are key contributors to healthy aging and have been shown to benefit physical, cognitive, and psychosocial health [30–33]. Although additional work is needed, joining a CAG may be an effective means of cultivating healthy aging among older adults.

Although the CAG members were generally positive in their feedback and description of their experience, they made two recommendations for working with citizen advisory group members in health program development research. First, one participant mentioned that it would be helpful for both citizen advisory group members and trainees to have prior training and preparation in writing about complex topics for a non-scientific audience. One member stated:

They were not given any instructions. So I think in the future there should be some preparation, it would be for everyone who’s going to be involved in writing. But if it’s not, if it’s the students, well, they should too, because it’s different writing for your thesis and writing for the general public. (CAG Member #3).

This highlights the importance of prior training in the knowledge translation domain when working with the community on research projects. Is it recommended that scientists and trainees leverage the expertise of and work with a knowledge translation practitioner from the outset of the project. Another recommendation was to provide citizen advisory group members closure at the end of the project by giving them access to view and interact with the final product:

It would be good to have the opportunity available. To look at the pilot project, to just to see how having worked on this for two years or three years or whatever, to see how the whole thing really flows. (CAG Member #2).

Providing the citizen advisory group with the opportunity to access the final product is important from a user-experience perspective and to thank them for their work.

While this study is important, it is not without several limitations. Namely, one of the CAG members did not participate in the focus group, rendering their opinions and experience missing from the data. Relatedly, only approximately one third of the chapter authors completed the survey. It is unknown if the authors that chose not to complete the survey differ in their perspectives on and experience working with the CAG. Moreover, we did not collect information about the chapter authors, beyond whether they were a trainee or a scientist. It would be worthwhile to know the authors’ sociodemographic backgrounds, fields of study, and prior experience with collaborative research approaches to better understand individual differences in perceptions of the CAG. It is also unclear how often the CAG members attended the optional chapter author meetings, which may have influenced their satisfaction with chapter author engagement. Finally, the original purpose of the CAG, in addition to the development of the program content, was to also advise on participant recruitment and program implementation. Given delays in the project, the CAG was unable to fulfill this objective. It would be interesting to know if their satisfaction and experience with this aspect of the program might have differed from the roles that they ended up carrying out.

## Conclusions

The BHPro CAG conferred benefits for older adult CAG members, chapter authors, and for the utility and accessibility of BHPro for end-users. The CAG’s objectives were met, and the inclusion of the CAG aligned with the guiding principles of integrated knowledge translation, co-production, engaged scholarship, and, to a lesser extent, participatory research. It is evident that collaboration with end-users through the involvement of a citizen advisory group provides unique insights in the development process that will improve the program’s accessibility, user experience, and uptake of brain-healthy behaviours and attitudes [28]. A citizen advisory group also directly impacts scientists, trainees, and citizen advisory group members alike. Future research should consider representation of the end-user in the composition of a citizen advisory group, as well as anonymous communication among citizen advisory group members and scientists/trainees during the development process to ensure equitability in power dynamics. It may also be beneficial to explore the utility of a knowledge translation practitioner’s support in mediating and facilitating the implementation of citizen advisory group feedback. Overall, this study highlights the benefits of collaborative research between researchers and end-users in the development of health and educational interventions.

## Supplementary Information

Additional file 1.

## Data Availability

The data that support the findings of this study are not publicly available due to their containing information that could compromise the privacy of research participants but are available from the corresponding author upon reasonable request.

## Abbreviations

BHPro: Brain health PRO
CAG: Citizen advisory group
