# Supplementary material for: Engaging older adults in the process of aging research: a multimethod study evaluating the experience and efficacy of a citizen advisory group for a dementia risk reduction program
Source: Res Involv Engagem. 2024 Dec 27;10:135. doi: 10.1186/s40900-024-00643-6 (PMC11674478; doi:10.1186/s40900-024-00643-6)
Supplement: Supplementary file 1 — Additional file 1. [file 40900_2024_643_MOESM1_ESM.docx]

**Supplementary Material**

Supplementary Table 1*. GRIPP2 Short Form for the current study.*

| Section and topic | Item | Reported on page No |
| --- | --- | --- |
| 1: Aim | Report the aim of PPI in the study | 6-7 |
| 2: Methods | Provide a clear description of the methods used for PPI in the study | 9-11 |
| 3: Study results | Outcomes – Report the results of PPI in the study, including both positive and negative outcomes. | 12-18, Figures 1-3 |
| 4: Discussion and conclusions | Outcomes – Comment on the extent to which PPI influenced the study overall. Describe positive and negative effects. | 18-24 |
| 5: Reflection/critical perspective | Comments critically on the study, reflecting on the things that went well and those that did not, so others can learn from this experience. | 18-24 |

*Note*. PPI: patient and public involvement

Supplementary Table 2. *Survey questions administered to the BHPro chapter authors and CAG members to assess the degree to which the CAG met it’s intended objectives.*

| Survey Question |
| --- |
| 1. Advising on the overall plan of BHPro (e.g., objectives, scheduling, time demands on BHPro participants) |
| 1. Reviewing the material and providing feedback on the individual chapters of the BHPro |
| 1. Assessing whether the chapters accomplish their stated intention/objective |
| 1. Assessing whether the information in the chapters was clear and understandable |
| 1. Assessing if the language of the chapters was appropriate to the intended audience |
| 1. Assessing if the chapters encouraged specific actions for older adults to promote brain health |
| 1. Assessing if the specific actions encouraged were realistic |
| 1. Advising on the implementation of the BHPro |
| 1. Advising on methods and sources of participant recruitment for the BHPro |

*Note.* BHPro: Brain Health PRO; Each statement was rated from 1 (*strongly disagree)* to 5 (*strongly agree*)

Supplementary Table 3. *Survey questions administered to the BHPro chapter authors and CAG members to assess how inclusion of the CAG aligned with collaborative research approaches.*

| Collaborative Research Approach | Survey Question |
| --- | --- |
| Integrated knowledge translation | 1. The BHPro scientists and the CAG worked in a collaborative manner |
|  | 1. The approach between BHPro scientists and the CAG will increase the chances that the research findings will be applicable to community-based older adults |
|  | 1. BHPro will increase knowledge on dementia prevention and brain health promotion among community-based older adults |
|  | 1. BHPro will improve the uptake of brain-healthy behaviours among community-based older adults |
| Integrated knowledge translation &  Participatory research | 1. The BHPro scientists and the CAG were treated as equals through the entire development process |
| Engaged scholarship | 1. The unique perspectives of the CAG were considered in the development process |
|  | 1. BHPro will advance both knowledge and practice in regard to dementia prevention |
|  | 1. The CAG made BHPro scientists aware of the dementia prevention needs of older adults |
|  | 1. Expertise from both the BHPro scientists and the CAG was leveraged during the development process |
| Co-production | 1. The CAG was actively involved in all stages of the development process |
|  | 1. The involvement of the CAG in the development process will lead to enhanced effectiveness of BHPro |
|  | 1. Compared to traditional approaches where research is carried out by scientists only, the CAG provided an opportunity for older adults to be more involved in the planning and delivery of BHPro |
| Participatory research | 1. The relationship between BHPro scientists and the CAG fostered a common understanding of ways to promote brain health with aging and prevent dementia |
|  | 1. The lived experience and knowledge of the CAG was mobilized in the development of BHPro |
|  | 1. The relationship between BHPro scientists and the CAG created a sense of shared ownership of BHPro |
|  | 1. BHPro will benefit underserved/vulnerable communities |
|  | 1. BHPro will meet the unique needs of underserved/vulnerable communities |
|  | 1. The CAG had equal or equitable power throughout the development process |
|  | 1. The CAG had an equal or equitable authority throughout the development process |

*Note.* BHPro: Brain Health PRO, CAG: Citizen Advisory Group; Each statement was rated from 1 (*strongly disagree)* to 5 (*strongly agree*)

Supplementary Table 4. *Survey questions administered to the CAG members only to gain a more in-depth understanding of their satisfaction with their involvement in the project.*

| Objective | Survey Question |
| --- | --- |
| Personal experience as a CAG member | 1. Overall, my personal learning objectives for being a CAG member were met |
|  | 1. The supports I needed to participate in the CAG and do a good job were made available and accessible to me |
|  | 1. A sense of collaboration and community was fostered among the CAG members |
|  | 1. My opinions and input were considered when decisions were made |
|  | 1. The time commitment for being a CAG member was consistent with my expectations |
|  | 1. I found the work to be intellectually stimulating |
|  | 1. I learned something new through my involvement in the CAG |
|  | 1. I would recommend involvement in the CAG to a friend |
|  | 1. My unique expertise and perspective contributed to the success of the CAG |
|  | 1. My involvement in the CAG will make a meaningful and positive impact on research in brain health and risk reduction for dementia |
|  | 1. I felt that my views were heard, valued, and respected by the scientists who authored the chapters |
|  | 1. If there were differences in opinion or disagreements with the scientists who authored the chapters, it was handled appropriately |
|  | 1. A sense of collaboration and community was fostered with the scientists who authored the chapters |
| Experience during expert team meetings | 1. I had enough information to contribute to the topics being discussed |
|  | 1. I felt confident and comfortable contributing to discussions |
|  | 1. I felt comfortable bringing up my concerns and frustrations |
|  | 1. My concerns and frustrations were addressed appropriately |
|  | 1. I had the opportunity to express my opinions |
|  | 1. I felt that my views were heard, valued, and respected |
|  | 1. I found these meetings beneficial for understanding the objectives and progress of BHPro |
| Experience during meetings with other CAG members | 1. I had enough information to contribute to the topics being discussed |
|  | 1. I felt confident and comfortable contributing to discussions |
|  | 1. I felt comfortable bringing up my concerns and frustrations |
|  | 1. My concerns and frustrations were addressed appropriately |
|  | 1. I had the opportunity to express my opinions |
|  | 1. I felt that my views were heard, valued, and respected |
|  | 1. If there were differences in opinion or disagreements between the CAG members, it was handled appropriately |
| Satisfaction with CAG protocols and parameters | 1. The overall scope of the CAG in terms of intended objectives and terms of reference |
|  | 1. The frequency of meetings |
|  | 1. The duration of meetings |
|  | 1. The overall leadership of the group (example: chairing meetings, coordinating roles) |
|  | 1. The overall management of the group (example: scheduling meetings, email communication) |
|  | 1. Engagement with other CAG members |
|  | 1. Engagement with the scientists who authored the chapters |
|  | 1. The amount of time provided to review BHPro chapters |
|  | 1. The ease of use of the chapter review document |
|  | 1. Follow up and communication after meetings |

*Note.* BHPro: Brain Health PRO, CAG: Citizen Advisory Group; Questions 1-27 were rated from 1 (*strongly disagree)* to 5 (*strongly agree*); Questions 28-37 were rated from 1 (*very dissatisfied*) to 5 (*very satisfied*)

**CAG Focus Group Interview Script**

1. Overall, how would you describe your experience as a CAG member?
2. How would you describe your experience working with the scientists who authored the chapters?
   1. Can you provide an example of how your unique perspectives were considered throughout the process?
   2. In what ways could your engagement with the authors have been improved?
      1. How do you think the process could be changed to make the power between the CAG and the authors more equal/equitable?
      2. In your opinion, what changes could be made to make you feel more comfortable/confident in contributing to discussions with the scientists?
3. Can you provide an example(s) of how the unique approach between the CAG and the scientists will increase the chances that Brain Health PRO will be applicable to older adults in the community?
4. Do you think Brain Health PRO would help *you* make healthy lifestyle changes to support your brain health and reduce dementia risk? Why or why not?
   1. Do you think Brain Health PRO will be effective at reducing dementia risk in the general Canadian population? Why or why not?
   2. How do you think the CAG has made a positive impact on the effectiveness of Brain Health PRO?
5. How could Brain Health PRO be modified to better benefit underserved/marginalized communities?
6. Is there any aspect of Brain Health PRO that you wish the CAG was more directly involved in? Why or why not?
7. Thinking about the make-up of the CAG membership, are there any lived experiences that you think were missing?
   1. Would it have helped if we had representation from different work experiences? Areas of Canada? Educational backgrounds? Racial/ethnic backgrounds? LGBTQ+ membership? People with disabilities?
8. Is there anything else you would like to comment on about the CAG and its work with Brain Health PRO?
